# *Salvia miltiorrhiza* Injection Ameliorates Renal Damage Induced by Lead Exposure in Mice

**DOI:** 10.1155/2014/572697

**Published:** 2014-02-13

**Authors:** Lei Li, Yuanyuan Zhang, Juanjuan Ma, Weichong Dong, Qiongtao Song, Jianping Zhang, Li Chu

**Affiliations:** ^1^Department of Pharmacology, School of Basic Medicine, Hebei Medical University, 326 Xinshi South Road, Shijiazhuang, Hebei 050091, China; ^2^Department of Internal Medicine, Baoding First Hospital of Traditional Chinese Medicine, Yuhua Western Road, Baoding, Hebei 071000, China; ^3^Department of Pharmaceutical Analysis, School of Pharmacy, Hebei Medical University, 361 Zhongshan East Road, Shijiazhuang, Hebei 050017, China

## Abstract

Exposure to lead (Pb) can induce kidney injury and our recent studies have found that *Salvia miltiorrhiza* (SM) injection, a traditional Chinese medicine, could protect against the organ injury induced by iron overload. This study was designed to investigate the protective effects of SM injection on nephrotoxicity induced by Pb acetate in mice and to elucidate the potential mechanism(s). Healthy male mice were randomly divided into four groups: control, Pb, low-dose *Salvia miltiorrhiza* (L-SM), and high-dose *Salvia miltiorrhiza* (H-SM). SM injection dose dependently reduced the Pb accumulation in the kidney, decreased kidney coefficients, and ameliorated renal structure and function from the morphology analysis. Meanwhile, SM administration downregulated serum levels of blood urea nitrogen (BUN) and creatinine (CR), decreased malondialdehyde (MAD) content, and increased activities of super oxide dismutase (SOD) and glutathione peroxidase (GSH-Px) in the kidney homogenate. Moreover, SM injection reduced the level of renal apoptosis by immunohistochemical staining analysis. Our findings implicate the therapeutic potential of SM injection for Pb-induced nephrotoxicity, which were at least partly due to the decrease of Pb accumulation, inhibition of lipid peroxidation, and suppression of renal apoptosis. These results provided preliminary experimental support for Danshen as a therapeutic drug for Pb poisoning diseases.

## 1. Introduction

Lead (Pb) is a common environmental contaminant and excessive exposure to it may cause chronic or acute nephrotoxic effects. Acute Pb nephropathy, mainly reported in children, is characterized functionally by a generalized deficit of tubular transport mechanisms (Fanconi syndrome) and morphologically by the appearance of degenerative changes in the tubular epithelium and nuclear inclusion bodies containing Pb protein complexes [1]. Pb poisoning mainly inhibits cell enzymes that contained thiol and leads to the body's biochemical and physiological dysfunction, causing small artery spasm and capillary endothelial cell injury, changing the properties of the normal erythrocyte membrane, inhibiting muscle phosphocreatine resynthesis, and so forth. Thus this gives rise to a series of pathological changes, wherein the nervous system, the kidneys, and hematopoietic system are more remarkable.

Clinical trials and epidemiological studies have demonstrated that lead poisoning can cause kidney damage. Currently, chelating agents such as disodium edetate calcium (EDTANa_2_Ca) and sodium dimercaptosuccinate (NaDMS) are commonly used in clinical to treat lead poisoning. Chelating agents form an insoluble complex with Pb for removal from the Pb-burdened tissue, but they are incapable of removing metal from intracellular sites and may cause redistribution of the toxic metal, essential metal loss, and liver or renal dysfunction [2].

*Salvia miltiorrhiza* (SM), commonly known as Danshen, has been used for hundreds of years in China and other countries. SM injection, the aqueous extracts of SM, has been widely and successfully used in the treatment of cardiovascular diseases and renal failure [3]. Recent studies have highlighted the diversity of potential effects of SM in attenuating microcirculatory disturbances, including antioxidation, inhibition of apoptosis, and amelioration of injury to target organs such as the kidney [4]. And our recent studies have found that SM injection could protect against cardiac damage, liver injury, hepatic fibrosis, and kidney injury induced by acute or chronic iron overload [5–8]. So we deduced that SM may have a protective effect on the renal damage induced by divalent heavy metal poisoning.

Voltage-dependent calcium channels found in a variety of cells are blocked by different metal cations. Pb^2+^ has been shown to perturb Ca^2+^-mediated cellular processes [9]. Pb^2+^ mimics Ca^2+^ in many biochemical and physiological processes and also competes with Ca^2+^ for membrane channels to gain entry into the cell [10]. Our recent study indicated that calcium channel blockers (CCBs) verapamil and nimodipine may have protective effects on nephrotoxicity induced by Pb acetate in mice [11]. Consequently, inhibiting Ca^2+^ channels has become a potential therapeutic approach for reducing kidney injury induced by Pb exposure. According to previous research findings, salvianolic acid B, one of the main active components in SM injection, has the capability of reducing the production of oxygen free radicals [12] and inhibiting Ca^2+^ channels [13]. In addition to the antioxidation and antiapoptosis, SM may act as a CCB to fight against renal injury induced by Pb poisoning.

In view of the importance of Pb intoxication in the pathological process of renal disease, it needs new drugs to treat Pb poisoning. Therefore, this study investigated the SM injection to protect against Pb nephrotoxicity in mice and the potential mechanisms.

## 2. Materials and Methods

### 2.1. Animals and Drugs

Eighty male Kunming mice weighing 20.0 ± 3.0 g were purchased from the Experimental Animal Center, Hebei Medical University, and housed in plastic cages with well-ventilated stainless steel grid tops at controlled temperature with a 12 h light-dark cycle. Animals were allowed to access normal chow and drinking water *ad libitum*. All animal handling procedures were in accordance with the Guidelines of Animal Experiments from the Committee of Medical Ethics, Ministry of Health of China, and experiments were approved by the Ethics Committee for Animal Experiments of Hebei Medical University (approval number: HEBMU-2011-09, approval date: September 07, 2011).

SM injection, which was approved by the State Food and Drug Administration (approval number: Z32020161), was obtained from Shenlong Pharmaceutical Co., Ltd. (Jiangsu, China, batch number: 11040314). Pb acetate (analytically pure) was obtained from Kangpuhuiwei Technology Co., Ltd. (Beijing, China). The reference substances (purity ≥ 98%) for HPLC-UV: danshensu, protocatechuic aldehyde, and salvianolic acid B were purchased from the Tauto Biotech Co., Ltd. (Shanghai, China). Unless otherwise stated, other chemical reagents were obtained from Sigma-Adrich Inc. (St. Louis, MO).

### 2.2. Experimental Design

After one week of acclimation, all mice were randomly divided into four groups (*n* = 20 in each group): control, Pb, low-dose *Salvia miltiorrhiza* (L-SM), and high-dose *Salvia miltiorrhiza* (H-SM). Mice of the L-SM and H-SM groups were given i.p. injections of SM at 3 and 6 g·kg^−1^ per day (calculated according to the content of SM, 1.5 g·mL^−1^), respectively. Meanwhile, mice of the control group and Pb group received isovolumic saline by the same route. Mice of the Pb, L-SM, and H-SM groups were intraperitoneally (i.p.) injected with Pb acetate 40 mg·kg^−1^ per day four hours later on the same day. Furthermore, the normal saline (NS) was used to dilute the SM before injection. The entire experimental period lasted for 10 days, and all mice were maintained on normal chow and drinking water *ad libitum.* Food intake and activities of all mice were observed carefully every day, and body weight was measured at the beginning and end of the experiment. After recording the final body weight on the 10th day of the experiment, each animal was anesthetized with sodium pentobarbital (50 mg·kg^−1^). Blood was taken from femoral arteries and the serum was separated for further analysis. Subsequently, the kidneys were quickly excised and stored in the 4% paraformaldehyde solution or cryopreserved at −80°C.

### 2.3. Main Constituent Analysis of SM Injection

**Figure 1:**
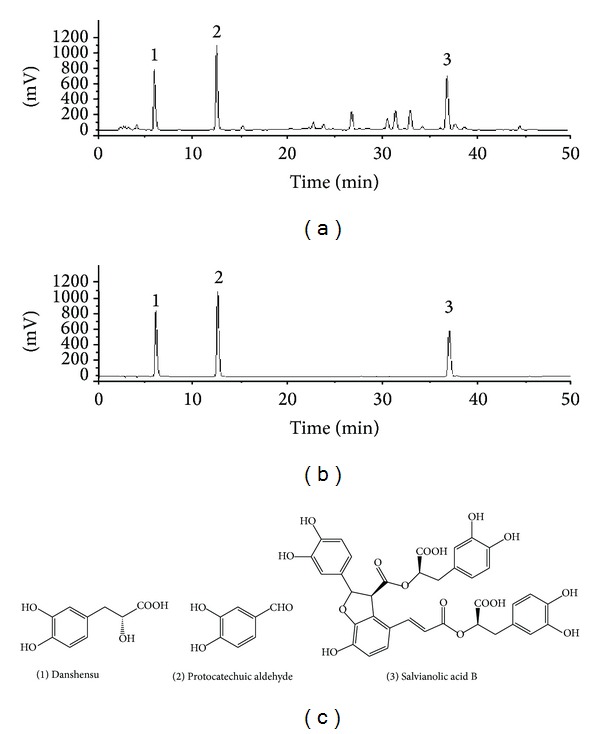
HPLC-UV profiles of SM injection, standards, and chemical structures of the active ingredients. HPLC-UV profiles of SM injection (a) and standards (b), and the peaks represented (1) danshensu; (2) protocatechuic aldehyde; (3) salvianolic acid B. (c) The chemical structures of the three active ingredients.

The main constituents of the SM injection were detected by high performance liquid chromatography with ultraviolet (HPLC-UV) and the chromatograms were presented in Figure 1. The conditions and procedures have been described previously [5].

### 2.4. Determination of Kidney Coefficients

Kidneys were removed and weighed using an electronic balance. The kidney coefficients were calculated as the kidney to body mass ratio × 100.

### 2.5. Tissues Pb Measurement

Pb concentrations in kidney homogenate were analyzed by flame atomic absorption spectroscopy (FAAS). Processing of the samples was performed as previously described [14]. The frozen kidney homogenate samples were dried at 65°C for 24 h. Dried samples were then weighed and ashed at 500°C for 12~16 h in a muffle furnace (Isotemp, Thermo Fisher Scientific, ON, USA). Ashed samples were digested by heating to a slow boil in 1 mL of concentrated hydrochloric acid (10 M, Thermo Fisher Scientific) and 3 mL of concentrated nitric acid (6 M, Thermo Fisher Scientific) over 4~6 h. One to two drops of hydrogen peroxide (H_2_O_2_, 4% w/v) were then added, and samples were boiled until only 0.5 mL of liquid remained. Water was added to concentrated samples (15~20 mL), and absorbance readings were performed at 283.3 nm using FAAS (Spectra AA-10 Varian Spectrophotometer). Standard curves for Pb were prepared from commercially available standards (Sigma). BSA 1577A and Tort-2 (National Institute of Standards and Technology, MD, USA) were used as controls for tissue digestion and preparation procedures.

### 2.6. Hematoxylin and Eosin (H&E) Examination

Four-micron sections were cut from paraformaldehyde fixed tissue embedded in paraffin blocks, and then the sections were dewaxed in xylene, rehydrated in descending alcohols, and washed in distilled water. All samples were stained with hematoxylin and eosin (H&E) for histological observations according to the manufacturer's protocol.

### 2.7. Assessment of Oxidative Stress and Lipid Peroxidation Indicators

Activities of glutathione peroxidase (GSH-Px) and super oxide dismutase (SOD) and concentration of malondialdehyde (MAD) were detected for assessment of the antioxidant capacity of SM injection. Each kidney tissue sample was weighed to prepare a 10% (w/v) buffered homogenate (100 mg tissue·mL^−1^ of 50 mM phosphate buffer, pH 7.2). The homogenates were centrifuged, and the supernatant was used for biochemical analyses. The protein concentration of the supernatant was determined by the Lowry method using bovine serum albumin (BSA) as a standard. The activities of GSH-Px and SOD as well as the production of MDA in kidney tissue were analyzed using a spectrophotometer and commercially available kits (Jian Cheng Biological Engineering Institute, Nanjing, China).

### 2.8. Assessment of Biochemical Indexes

Blood urea nitrogen (BUN) and creatinine (CR) were measured as biochemical indexes for assessment of the renal function. Levels of BUN and CR in serum were evaluated by spectrophotometry-based methods using commercially available kits (Jian Cheng Biological Engineering Institute, Nanjing, China).

### 2.9. Terminal Deoxynucleotidyl Transferase-Mediated dUTP Nick End Labeling (TUNEL) Staining

TUNEL staining was performed using an In Situ Cell Death Detection Kit (Roche Basel, Switzerland) according to the manufacturer's protocol. Briefly, the kidney sections were deparaffinized, dehydrated by a series of successively increasing concentrations of alcohol, washed in distilled water followed by PBS, and deproteinized by proteinase K (20 *μ*g·mL^−1^) for 30 min at 37°C. Then, the sections were rinsed and incubated in the TUNEL reaction mixture. After rinsing, sections were visualized using a converter-POD with 0.02% 3,3-diaminobenzidine (DAB). The sections were counterstained with hematoxylin. The number of tubular cells undergoing apoptosis was counted in three kidney sections of each animal, and the results were expressed as the average number of TUNEL-positive cells/mm^2^ ± SD. All counting procedures were performed in a blinded fashion.

### 2.10. Statistical Analysis

Data were expressed as mean ± SD. All data were analyzed statistically using analysis of variance (ANOVA), followed by Tukey's multiple comparison test. Statistical significance was considered at *P* < 0.05. The Statistical Package for Social Sciences (SPSS, Chicago, IL, USA) for Windows version 15.0 software was used for all analyses.

## 3. Results

### 3.1. Contents Analysis of SM Injection

The main constituent of SM injection was detected by HPLC-UV (Figures 1(a) and 1(b)). By calculation, the actual concentrations of the three active ingredients were danshensu 2.15 mg·mL^−1^, protocatechuic aldehyde 0.44 mg·mL^−1^, and salvianolic acid B 1.01 mg·mL^−1^. Furthermore, the chemical structure is shown in Figure 1(c).

### 3.2. Effects of SM Injection on Body Weight and Kidney Coefficients

The changes of body weight and kidney coefficients are shown in Table 1. The body weight gain of Pb-exposed mice was lower than that of the control group (*P* < 0.05). The body weights were decreased but the kidney weights were increased in the Pb group because the Pb accumulated in the kidney and injured the structure and function of the kidney. By the SM injection treatment, the body weight of mice in both L-SM and H-SM groups was higher and the kidney coefficients were lower than the Pb group (both *P* < 0.05).

### 3.3. Effects of SM Injection on Concentrations of Tissues Pb

**Figure 2:**
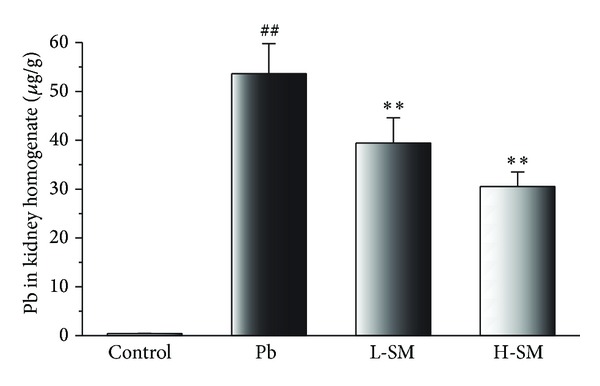
Effects of SM injection on concentrations of Pb in kidney homogenate. Samples were obtained from the control group, Pb group, L-SM group, and H-SM group. Data are expressed as mean ± SD of 20 mice. ^##^ *P* < 0.01, compared with control group; **P < 0.01, compared with Pb group.

As shown in Figure 2, the i.p. injection of Pb acetate resulted in a dramatic increase of tissue Pb concentrations. By contrast, SM treatment could significantly decrease the elevated level of Pb in the kidney homogenate (*P* < 0.01). This phenomenon explains that the increase of kidney coefficient is due to a lot of lead deposition in the kidney and SM could reduce the accumulation.

### 3.4. Morphological Changes Observed by H&E Staining

**Figure 3:**
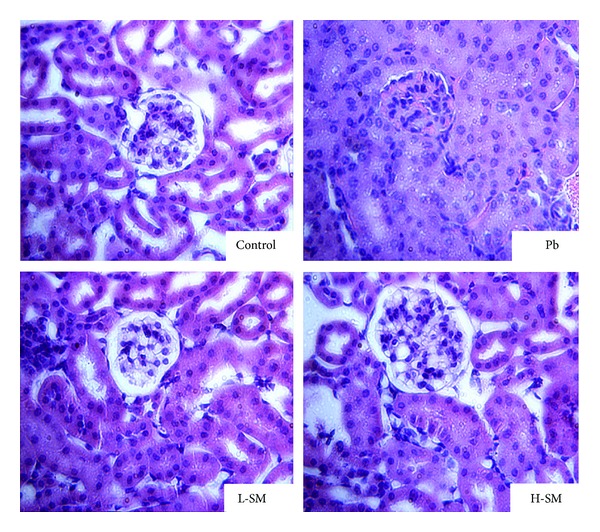
Effects of SM injection on histopathological changes stained with H&E. Representative microscopic photographs of kidney stained with H&E (magnification 400x). Compared to the normal morphology of kidneys in the control group, those of mice treated with Pb acetate displayed marked histological changes in the cortex and remarkable swelling in the proximal tubular epithelial cells, as well as diminishing glomeruli of the kidney. Although SM-treated mice showed some enlargement of proximal tubular epithelial cells, the glomerulus architecture was normal compared with the Pb group.

In the Pb treated mice, pathology slices showed the swelled renal tubule epitheliums and the atrophied glomerulus (Figure 3(b)). Although mice in the L-SM and H-SM treatment groups (Figures 3(c) and 3(d)) showed some enlargement of the renal tubule epitheliums, the glomerulus's architecture was normal compared with the Pb group (Figure 3(a)). The results indicated that excessive lead exposure can affect the structure and function of the kidney and the SM injection could ameliorate the injury of the kidney.

### 3.5. Effects of SM Injection on Tissue SOD, GSH-Px, and MDA

**Figure 4:**
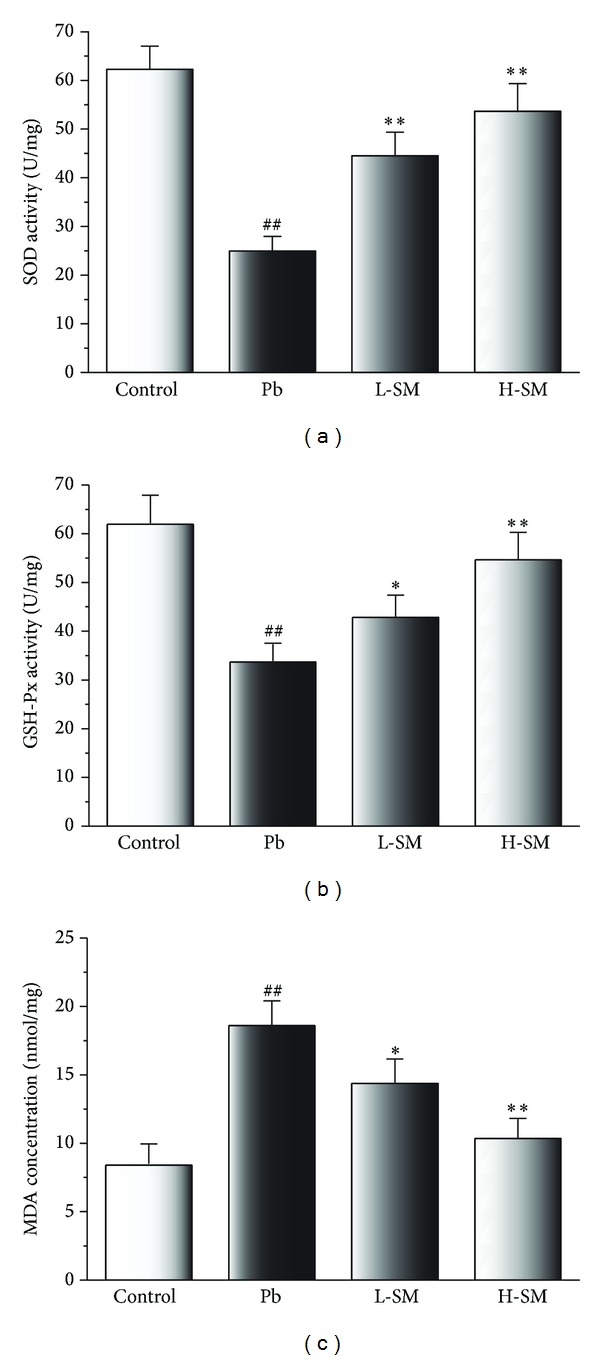
Effects of SM injection on activities of SOD, GSH-Px, and on the concentrations of MDA. Samples were obtained from the kidney homogenate of the experimental mice. Data are expressed as mean ± SD of 20 mice. ^##^ *P* < 0.01, compared with control group; *P < 0.05, compared with Pb group; **P < 0.01, compared with Pb group.

Compared to the control group, activities of SOD and GSH-Px were dramatically decreased in the Pb group (*P* < 0.01), while the activities of SOD and GSH-Px in the L-SM and H-SM groups increased (*P* < 0.01) (Figures 4(a) and 4(b)). Furthermore, the concentration of lipid peroxidative product MDA was significantly enhanced in the Pb group compared with the control group (*P* < 0.01), while L-SM and H-SM treatment markedly reduced the concentration of MDA (*P* < 0.01 and *P* < 0.05) (Figure 4(c)). These results demonstrated that SM treatment could protect against lipid peroxidation in the kidney induced by Pb exposure and the effect of high dose was better than the low dose.

### 3.6. Effects of SM Injection on Serum BUN and CR

**Figure 5:**
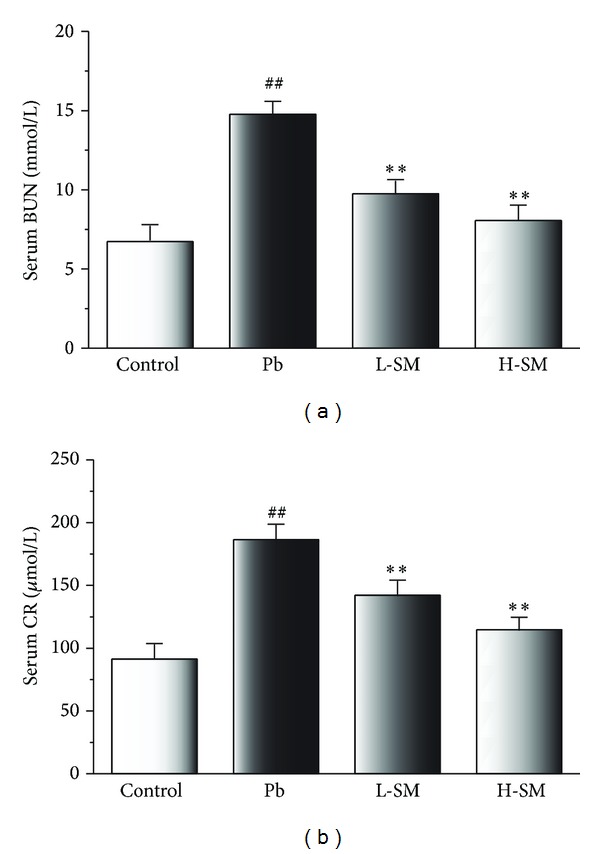
Effects of SM injection on serum concentrations of BUN and CR. Samples were obtained from the control group, Pb group, L-SM group, and H-SM group. Data are expressed as mean ± SD of 20 mice. ^##^ *P* < 0.01, compared with control group; **P < 0.01, compared with Pb group.

Compared with control mice, BUN and CR levels were notably elevated in the Pb-exposed animals (*P* < 0.01); meanwhile, serum BUN and CR levels were clearly lower in the SM-treated groups compared with the Pb group (*P* < 0.01) (Figure 5). These results indicated that SM injection could ameliorate the diminished renal function induced by Pb exposure.

### 3.7. Effects of SM Injection on Renal Apoptosis

**Figure 6:**
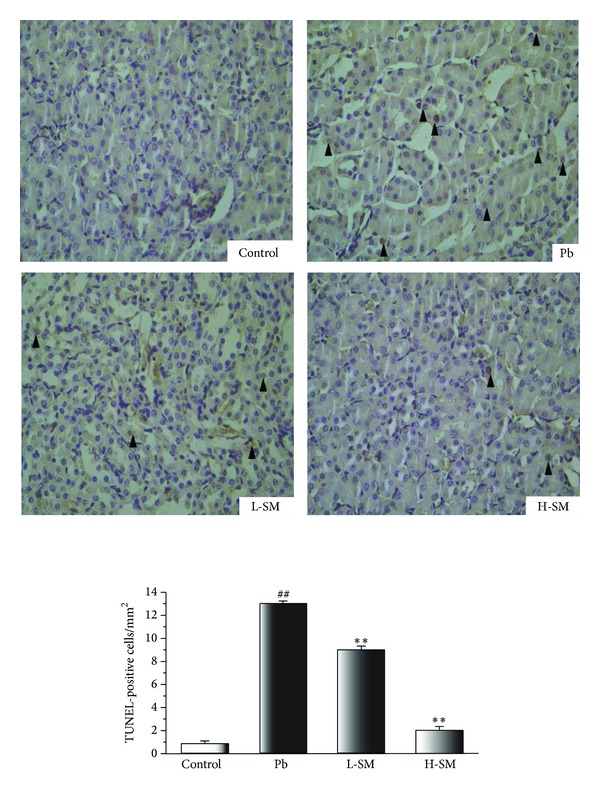
Effects of SM injection on the apoptosis of mouse kidneys. Representative microscopic photographs of TUNEL-stained kidney sections were shown (magnification 400x). Very few tubular and interstitial cells were TUNEL-positive in the control group. The number of apoptotic cells markedly increased with lead acetate injection. Both L-SM and H-SM treatments significantly decreased the number of TUNEL-positive cells in the kidney; the arrow heads pointed out the apoptotic cell nucleus. The histogram showed the relative proportion of TUNEL-positive cells in the kidney of mice. Data are expressed as mean ± SD of 20 mice. ^##^ *P* < 0.01, compared with control group; **P < 0.01, compared with Pb group.

The extent of tubular cell apoptosis remained at a constantly low level in the control mice, while of TUNEL-positive cells was dramatically raised in the Pb group and the proportion was decreased to some extent in L-SM and H-SM groups (Figure 6). These results demonstrated that SM injection treatment could suppress the level of renal apoptosis induced by Pb exposure.

## 4. Discussion

Lead (Pb) is one of the ubiquitous heavy metal poisons in the environment. The pathogenesis of Pb exposure is multifaceted, including the interruption of enzyme activity and protein function by directing binding to the sulfhydryl group, induction of free radicals, depletion of antioxidant reserves, and the competitive inhibition of mineral absorption and disturbance of homeostasis [15]. The kidney serves as a major organ of Pb excretion and accumulation and a sensitive target organ for Pb exposure. In recent studies, Pb has been shown to inhibit the activities of antioxidant enzymes, including glutathione peroxidase (GSH-Px), catalase, and super oxide dismutase (SOD) [16]. In this study, we have shown that mechanisms of Pb-induced kidney injury are related to increase in production of reactive oxygen species (ROS) and induction of oxidative stress and apoptosis.

SM injection containing bioactive compounds, such as salvianic acid A, protocatechuic aldehyde, and salvianolic acid B (Figure 1), has been used in clinical applications for years with no serious adverse reactions reported [3]. Some of the deleterious effects of Pb have been attributed to induction of oxidative stress, and Pb is known to disrupt the prooxidant/antioxidant balance of tissues that leads to biochemical and physiological disturbances [17]. As the commonly used Chinese herbal medicine SM can scavenge oxygen free radicals and inhibit lipid peroxidation, we hypothesized that it may be an effective treatment for kidney injury and therefore investigated its protective effects against renal damage induced by Pb exposure in mice.

In this investigation, the results demonstrated that SM injection could decrease the kidney coefficient (Table 1), reduce Pb accumulation (Figure 2), and ameliorate the histopathological changes in the kidney of Pb-exposed mice. Recent pharmacological studies have shown that salvianolic acid B could possibly facilitate the repair of tubular epithelial structures and the regression of renal fibrosis in injured kidney [18]. Furthermore, SM injection could inhibit the lipid peroxidation and decrease renal cell necrosis, thereby protecting the kidney from injury induced by Pb exposure. As Pb dosedependently accumulated in the mice, mostly in the kidney, the excessive Pb was clearly demonstrated to reduce body growth as well as increasing the kidney weight. However, the kidney coefficient was significantly decreased in all treated animals, indicating that the negative effects of Pb exposure in mice could be alleviated by SM injection administration.

Pb-induced toxicity was observed in the glomeruli and proximal tubules of the kidneys in the Pb group mice (Figure 3). Morphological changes indicated that SM treatment ameliorated the swelling in the renal tubules and the glomerular atrophy induced by Pb acetate. Danshensu has been demonstrated to inhibit calcium channels in vascular smooth muscle cells, which suggests that SM prevents the entry of Pb into the kidney tissue via negative effects on calcium channels [19].

Among the various pathogenic mechanisms of renal injury, oxidative stress alters glomerular structure and function mainly due to the effect of reactive oxygen species (ROS) on mesangial and endothelial cells [20]. Accumulating evidence shows that Pb causes oxidative stress by inducing the generation of ROS, which includs hydroxyl, superoxide radicals, and H_2_O_2_ [21]. Pb is not able to induce the production of free radicals directly, but it does influence indirectly the processes of lipid peroxidation, which may lead to tissue damage [22]. MDA, the main product of lipid peroxide that can seriously damage the cell membrane, is used as an indicator of cellular membrane damage caused by ROS [23]. SOD and GSH-Px are major enzymatic antioxidants that scavenge harmful ROS, and SOD also provides the first line of defense against free radicals by making toxic superoxide into less toxic H_2_O_2_ [24]. These antioxidant enzymes are potential targets for Pb toxicity [25]. With the increase in lipid peroxide levels in the body, activities of GSH-Px and SOD change accordingly. In this study, amounts of Pb accumulated in the kidney escalated the lipid peroxidation (increased MDA concentration) and oxidative stress (lowered SOD and GSH-Px activities) (*P* < 0.01, Figure 4). With low or high doses of SM injection treatments, animals exhibited lower levels of MDA and higher levels of SOD and GSH-Px than the Pb group mice (both *P* < 0.01). Thus, we concluded that SM injection significantly increased the renal antioxidant status by decreasing the content of MDA and increasing the activities of GSH-Px and SOD. The antioxidant properties of SM injection appeared to underlie the mechanism of protective effects against kidney injury.

The kidney has long been recognized as a critical target organ of Pb toxicity. The serum levels of BUN and CR can be interpreted as indices of renal dysfunction. The results showed that serum levels of BUN and CR dramatically increased (both *P* < 0.01) (Figure 5) in the Pb group. The increased levels of BUN and CR in the serum reflected deterioration of renal function. Importantly, we found that SM injection could significantly attenuate the elevation of serum BUN and CR levels, indicating that it effectively protected against kidney dysfunction.

Renal cell apoptosis was examined by TUNEL staining and a large number of apoptotic cells were found in the mice treated with Pb (Figure 6). SM injection administration effectively decreased the cellular apoptosis induced by Pb exposure in the kidney. Oxidative stress has been found to mediate age-associated renal cell injury and cell death, particularly apoptosis [25]. Furthermore, protocatechuic aldehyde has been reported to protect against TNF-*α*-induced endothelial cell injury by stimulating free radical scavenging activity and has been demonstrated to induce apoptotic cell death by inhibiting the ornithine decarboxylase activity [26].

In conclusion, this study demonstrated multitargeted protective effects of SM injection on renal damage in Pb-exposed mice by reducing the Pb accumulated in the kidney, suppressing lipid peroxidation, and inhibiting renal apoptosis. These findings provided preliminary experimental support of SM injection as a therapeutic drug for Pb poisoning diseases. As SM injection has a complex chemical composition and multiple pharmacological functions, a greater understanding of the mechanisms of SM injection may offer better strategies to protect against kidney dysfunction induced by Pb associated toxicity.

## Figures and Tables

**Table 1:** Effects of SM injection on body weight and kidney coefficients.

Groups	*n*	IBW (g)	FBW (g)	KW (g)	KC (%)
Control	20	20.28 ± 2.16	29.17 ± 2.56	0.31 ± 0.06	1.09 ± 0.25
Pb	20	20.72 ± 2.23	22.94 ± 3.07^#^	0.37 ± 0.07	1.78 ± 0.21^#^
L-SM	20	20.65 ± 1.72	25.36 ± 2.23*	0.32 ± 0.05	1.26 ± 0.16*
H-SM	20	19.99 ± 2.14	26.25 ± 3.21*	0.31 ± 0.08	1.18 ± 0.14*

IBW: initial body weight; FBW: final body weight; KW: kidney weight; KC: kidney coefficient.

Data are expressed as mean ± SD. ^#^ *P* < 0.05 compared with control group; *P < 0.05 compared with Pb group.

## Abbreviations

BUN: Blood urea nitrogen
CR: Creatinine
GSH-Px: Glutathione peroxidase
HPLC-UV: High performance liquid chromatography with ultraviolet
H-SM: High-dose *Salvia miltiorrhiza *
H&E: Hematoxylin and eosin
i.p.: Intraperitoneal
L-SM: Low-dose *Salvia miltiorrhiza *
MDA: Malondialdehyde
SOD: Superoxide dismutase
TUNEL: Terminal deoxynucleotidyl transferase-mediated dUTP nick end labeling.
